# Unravelling the Role of Chitin and Chitosan in Prebiotic Activity and Correlation With Cancer: A Narrative Review

**DOI:** 10.1093/nutrit/nuae168

**Published:** 2024-11-12

**Authors:** Irene Ferri, Benedetta Canala, Luciana Rossi

**Affiliations:** Department of Veterinary Medicine and Animal Sciences (DIVAS), University of Milan, Lodi 26900, Italy; Department of Veterinary Medicine and Animal Sciences (DIVAS), University of Milan, Lodi 26900, Italy; Department of Veterinary Medicine and Animal Sciences (DIVAS), University of Milan, Lodi 26900, Italy

**Keywords:** chitin, chitosan, edible insects, nutrition, prebiotic, cancer

## Abstract

This review describes the state of the art regarding the prebiotic role of chitin and the interactions of chitin and chitosan with cancer cells. Chitin is the second most abundant polysaccharide in nature and a constitutive component of crustacean shells and the exoskeleton of insects. Chitosan is the deacetylated form of chitin, which is obtained by chemical processing or the enzymatic activity of deacetylases found in microorganisms and insects. Edible insects have recently been introduced in Western countries, thus raising concerns regarding food safety and due to their chitin content and the release of chitosan during the digestive process. The roles of insect chitin and chitosan in the gastrointestinal tract, microbiome modulation, and cancer have been widely investigated. Several in vitro and in vivo studies have shown the possible microbiota modulation of chitin and its relevant communication with the immune system, thus confirming its prebiotic activity. No evidence has been provided on the cancerogenic activity of chitin; however, studies have suggested that chitin has a cytotoxic effect on cancer cell lines. Chitosan has been confirmed to exhibit apoptotic and cytotoxic activities on cancer cells in several in vitro studies on cancer cell lines and in vivo models. In conclusion, the literature does not show a direct connection between the presence of chitin or chitosan and the onset of cancer. However, cytotoxic and apoptotic activities in relation to cancerous lines have been demonstrated.

## INTRODUCTION

Edible insects are now considered to be an innovative and environmentally sustainable source of valuable nutrients, particularly protein. However, the consumption of insects around the world is associated with rich cultural traditions, beliefs, and gastronomy.^1^ In fact, in Asia, edible insects are commonly included in the diet. In some Asiatic markets, insects, such as worms, ants, and beetles, are regarded as popular street food. In Western countries, edible insects have been recently introduced, and their food safety and the ecological impact are being discussed.

Generally, insects have a high nutritional value because of their high content in essential amino acids, unsaturated fatty acids, and micronutrients (vitamin B_12_, iron, zinc, calcium).^2^^,^^3^ The growing interest in edible insects led the European Parliament and the Council of the European Union to declare insects as a novel food, which consists of any food destined for human consumption that was not used in large quantities in the European Union (EU) before May 15, 1997, and that has been included in any of the food categories established in the EU regulation 2283/2015. The European Food Safety Authority (EFSA) released a list of insect species that have a high potential for use in animal and human nutrition. Among these, EFSA regarded the following meals as being safe: *Tenebrio molitor* larvae, *Locusta migratoria*, and *Acheta domesticus* and *Alphitobius diaperinus.* Evaluating food safety is key before being able to market insects as food sources, as it takes into account the nutritional components of novel food.

With regard to insects, chitin is a widely discussed polymer. Chitin represents the prevalent fraction of insoluble fiber in insects and is the most widespread polysaccharide in nature after cellulose.^4^^,^^5^ It is thus the main component of an insect’s exoskeleton. Chitin could be enzymatically or physically modified into derivatives, of which chitosan is the most representative.^6^ Interest in investigating the health impact of chitosan is related to the wide scientific application of the molecule. Moreover, public concern has focused on the possible release of chitosan after the digestive process of chitin in monogastric animals. The role of these composites has been widely discussed, mainly in relation to gut interactions, microbiome modulation, and cancer. Several studies have also investigated the mechanisms of action of chitin in the gastrointestinal tract and the subsequent effects on the microbial population and cell cancer activity. This review is thus aimed at providing a detailed description of the state of the art of the prebiotic role of chitin and the interactions of chitin and chitosan with cancer cells.

## METHODS

For this narrative review, we searched PubMed and Scopus for all relevant articles published from 2011 up to 2023, using the following key terms: chitin, edible insect, microbiota, prebiotic, and cancer. The considered words were combined with the Boolean operators AND, OR. The identified articles were then classified, critically discussed, and summarized.

## CHITIN AND CHITOSAN: ORIGIN AND STRUCTURE

### Origin and Synthesis of Chitin

Chitin is a 2-acetamido-2-deoxy-β-d-glucopyranose polymer that is widespread among organisms. This molecule was isolated for the first time in 1811 from an alkali-resistant fungi by the chemist Henri Braconnot.^7^ In particular, chitin acts as support and physical barrier of the shell of crustaceans, the exoskeleton of insects, and as a component of the cell walls of fungi. However it has also been found in diatoms, zooplankton, and nematodes.^8^ In nature, cellulose is the carbohydrate most similar to chitin. The difference between them is the presence of the acetamide group in chitin, which is bonded to the carbon in the 2-position instead of the hydroxyl group.^9^ The resulting monomer is the N-acetyl-d-glucosamine. Chitin synthesis is performed by an enzyme located on the plasma membrane called chitin synthase, which belongs to the glycosyltransferase-2 (GT-2) family.^8^^,^^10^ Chitin synthase binds N-acetyl-d-glucosamine monomers by beta-1–4 glycosidic bonding to form linear carbon chains.

### Chitin Structure and Allomorphs

The chitin structure appears to be organized as a crystal and was thus analyzed in order to investigate the biological properties of the molecule in terms of its stereochemical characteristics. Crystallographic analyses have revealed alternative forms of chitin called allomorphs. Three types of molecules have been identified and differentiated into alpha, beta, and gamma.^11^ Recent studies have confirmed that the gamma allomorph is a different form of alpha, with very similar characteristics. The substantial differences between alpha and beta lie in the polarity and packaging with near chitin chains. All of the allomorphs are made up of piles of chitin chains that interact through carbonyl and amine groups from the N-acetyl side chains of glucosamine residues and hydrogen bonds between the sheets.^9^ The resulting carbon filaments therefore have different polarities.

Alpha chitin, the allomorph mainly found in the shells of crustaceans and the exoskeleton of insects,^12^ is characterized by alternating sheets with opposite binding polarities—that is, antiparallel. In contrast, beta chitin, which is typical of diatoms, annelid worms, and mollusks,^11^ has a parallel sheet arrangement. Gamma chitin, similar to alpha, has a mixed arrangement in which 2 sheets have parallel and 1 sheet an antiparallel direction.^13^ The analysis, study, and knowledge of the structural organization of chitin are crucial in order to evaluate the impact on animal and human health.

### Chitin Digestion, Chitinases, and Chitosan

Chitin digestion is performed by a class of enzymes called chitinases, which belong to the 18th and 19th families of glycoside hydrolases. This enzyme has been found in viruses, bacteria, and fungi to protect the microorganisms. The discovery of chitinase in mammals is recent, and it is thought to counteract exogenous chitin^8^; thus, basal levels of the enzyme may be dependent on specific conditions. For this reason, animals, especially monogastrics, show enzyme inefficiency for the digestion of soluble fibers including chitin. It is thus essential to know the chitin content of organisms.

**Figure 1.**
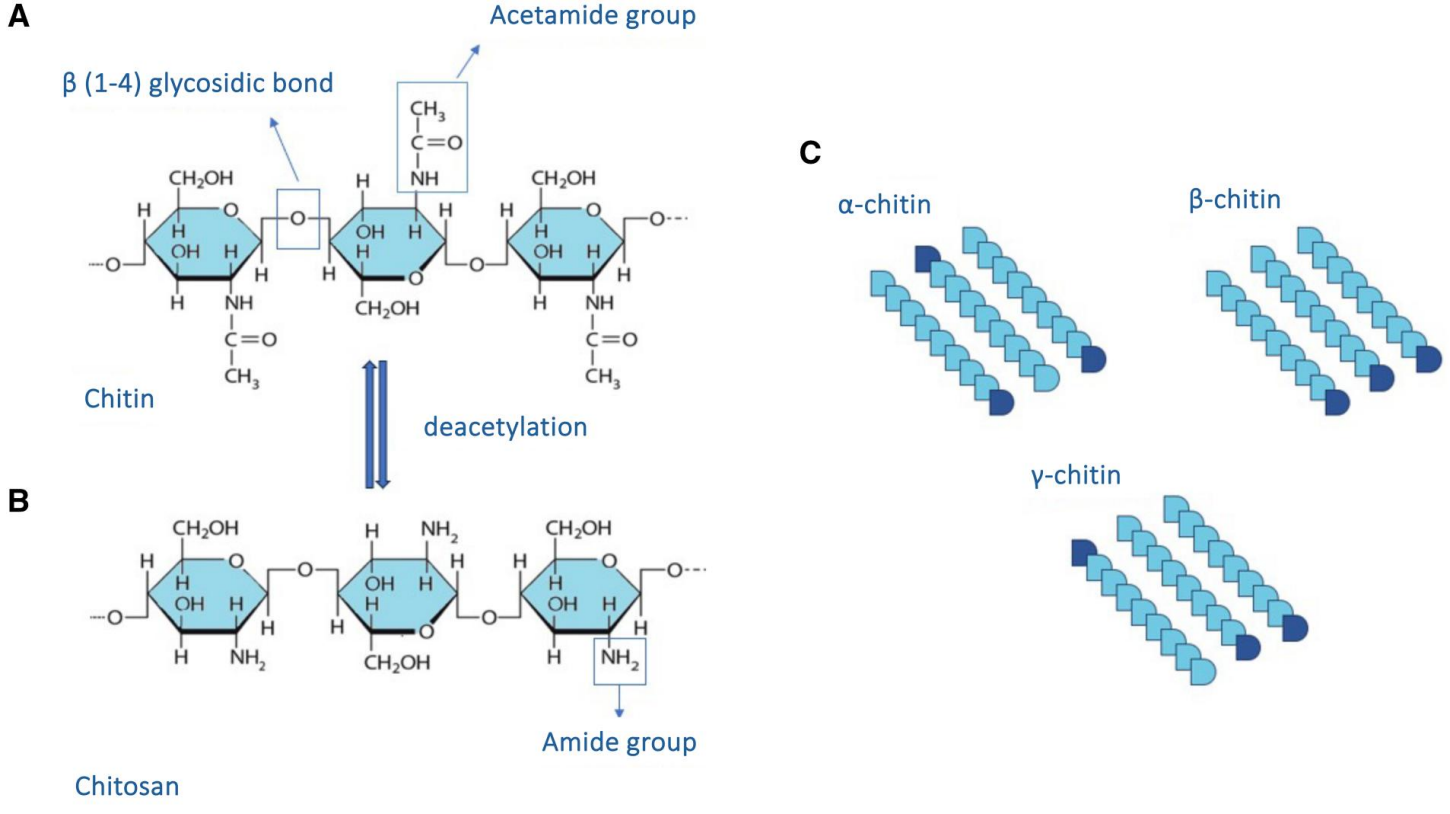
Chemical Structures of Chitin and Chitosan. (A) Chitin homopolymer of N-acetyl-d-glucosamine monomers bound by β-(1–4)-glycosidic linkages. (B) Chitosan structure as a result of deacetylation process. (C) Chitin allomorphs and the various polarities

With regard to chitin derivatives, chitosan is the deacetylated form, which consists of a polymer of 2-amino-2-deoxy-β-d-glucopyranose.^14^ Chitosan can be obtained by chemical processing using a strong base (NaOH) or by enzymatic activity of the deacetylase found in microorganisms and insects.^15^^,^^16^ In contrast to chitin, chitosan is a crystalline molecule with high hydrophilicity and with no intermolecular hydrogen bonds compared with the original polymer, thus resulting in increased viscosity (Figure 1). Many polysaccharides are neutral or negatively charged, whereas chitosan is a cationic carbohydrate due to the strong presence of glucosamine.^9^^,^^16^^,^^17^ The fact that chitosan is a cationic carbohydrate is not only a determining factor in intermolecular and cellular interactions but also in improving techniques for extracting and modifying the molecule in the laboratory.

## EFSA STATEMENT ON THE SAFETY OF INSECT CHITIN

### Chitin Content of Edible Insects

In the EU, the classification of insects as a novel food is crucial in the context of food safety and the availability of sustainable protein sources.^18^^,^^19^ The recognition of insects as potential food ingredients requires careful scientific evaluation to ensure regulatory compliance and public health protection.^20^

The EFSA has designated 4 insect species as safe for human consumption, for which the European Commission has subsequently given market approval.^21^ The introduction of edible insects in the diet has generated growing interest among consumers, but, in parallel, it has also raised some concerns. In particular, the focus has been on the role of chitin in human and animal nutrition. The chitin content varies according to the species, sex, and stage of development of the insect, and ranges from 2.35% (cricket) to 45% (*Bombyx eri*). *Acheta domesticus* (house cricket) and *Tenebrio molitor* (mealworm beetle), which are the most commonly used species in human and animal nutrition, have average chitin contents of 5.8% and 6.15%, respectively.^5^ These values tend to be lower than in other insect species, which is a positive aspect for nutrition. Based on several studies on edible insects, the EFSA considers chitin as a nutritional component, and is reported to be a dietary fiber.

### Safety Issues of Chitin From Insect-Derived Food Products

The greatest concerns of the EFSA regarding chitin are related to the influence on protein consumption and digestion and the bioavailability of minerals. Analysis of an insect’s protein content is based on a nitrogen conversion coefficient. The molecular structure of chitin consists of non-protein nitrogen; thus, in calculating the protein percentage of insect meal, the nitrogen conversion coefficient has to be modified.^22^ Chitin ingestion can also affect the bioavailability of some bivalent minerals through the generation of specific binding, as reported for dietary fiber in general.^23^

However, one of the important issues for human health is the correlation between chitin and the immune response.^24^ Generally, an allergic reaction is a defensive mechanism against pathogens. In the case of chitin, the immune system considers it a non–self-element, resulting in a wide spectrum of symptoms. However, the immune response occurs in predisposed individuals or those particularly exposed to massive allergen doses for a prolonged period.^25^ Although prolonged inflammatory conditions could contribute to cancer development,^26^ there is no scientific evidence of the direct induction of cancer by chitin ingestion. The EFSA therefore does not associate chitin with potential risks for human health. However, to ensure the safety of the product for human consumption, the EFSA suggests a range of chitin content in novel food that is considered “safe” and that cannot interfere with physiological digestive processes. Accepted values for chitin intake are determined by considering daily consumption patterns among the population's demographic factors, such as regional variations, age groups, or characteristics such as the type of product (eg, snacks are different from pasta consumption). This approach guarantees that chitin levels are not dangerous to the consumer, even in the hypothetical scenario of a significantly high product intake.

## DIETETIC CHITIN: PREBIOTIC ACTIVITY AND MICROBIOTA MODULATION

### Maintenance of Eubiosis and Health Status

Digestion is a complex process consisting of the passage, degradation, and intake of food bolus through designated organs. The microbiome plays a key role in the digestive process. The fermentative activity of the microbiome influences the systemic organs, resulting in microbiome–organ axes. Although the correlation between disease and microbiota composition is still not known, several studies have reported that there is a positive correlation between the richness and diversity of the microbiota and its fermentation products and health status.^27^ The composition of the microbiota can control the onset of various diseases, such as cardiovascular diseases, intestinal inflammation, diabetes, obesity, neurological disorders, and cancer.^28^

Colorectal cancer is one of the most common intestinal diseases. Its onset is often not attributable to a single cause but should be defined as a multifactorial disease. Prevention of colorectal cancer could also depend on microbiota. An imbalance regarding pathogenic microbial species such as *Escherichia coli*, *Fusobacterium nucleatum*, or *Bacteroides fragilis* can induce DNA damage and tumorogenesis.^29^ As a consequence, in a state of eubiosis, a positive balance between the different microbial populations and the environment needs to be maintained. Injury to the intestinal barrier could lead to severe inflammation or could be a symptom of cancer onset. In order to protect intestinal integrity, the microbiota modulates the immune response. However, the microbiota can also interact directly with the immune system through various mechanisms including Toll-like receptor (TLR) signaling and inflammasome sensing.^30^^,^^31^ Therefore, in order to preserve eubiosis, the supplementation of ingredients and/or microorganisms that can promote it is strongly recommended.

### Prebiotic Effect of Chitin

Prebiotics are defined as “undigestible food ingredients that beneficially affect the host by selectively stimulating the growth and/or activity of one or a limited number of bacterial species that are already established in the colon and therefore improve the health of the host.”^32^ Prebiotics preserve and improve the integrity of the intestinal barrier, which is the check-point for the entry of nutrients for the digestive/fermentative process and blocks pathogen growth–promoting substances. The intestinal barrier is thus strictly related to the immune system. As a nondigestible dietary fiber, chitin falls into the category of a prebiotic. Contrary to public concern regarding the possible negative effects of chitin intake, more recent in vivo studies and clinical trials have shown an unaltered general health status in healthy subjects (Table 1). In the clinical trial described by Rodriguez et al,^33^ volunteers reported no negative impacts on their physical and mental state throughout the entire trial period. In addition, no gastrointestinal tract disorders or reductions in quality of life were detected. Prebiotic chitin does not alter the state of the intestinal barrier^34^^,^^35^ and helps to maintain the richness and relative abundance of the microbial species that constitute the microbiota.^36^

**Table 1.** In Vivo and In Vitro Studies on the Prebiotic Activity of Chitin and Chitosan From 2017 to 2020

Molecule and assay	Study	Source	Activity	Description	Reference
Chitin					
In vivo	Study on inclusion of insects as protein source in poultry diet	Insects (*Hermetia illucens*)	Prebiotic activity	Soybean meal substituted by insect meal; 1.02 g/d of chitin	Borrelli et al (2017)^34^
	Impact of chitin–glucan complex	NR	Prebiotic activity	Chitin–glucan (2-5 g/d, 3 wk) promotes *Roseburia* and butyrate production	Rodriguez et al (2020)^33^
In vitro	In vitro digestion of *Tenebrio molitor* meal and human feces fermentation	Insects (*Tenebrio molitor*)	Prebiotic activity	Chitin improves Bacteroidates and Prevotellaceae strains, inhibits *Clostridium* histolyticum, Desulfovibrionales, and Desulfuromonales; chitin promotes SCFA production	de Carvalho et al (2019)^40^
	Evaluation of 10%-20% and 39% of partial substitution of *Hermetia illucens* in rainbow trout diets (12 wk)	Insects (*Hermetia illucens*)	Prebiotic activity	Chitin improves Actinobacteria and Proteobacteria phyla, improves microbiome richness, and promotes SCFA production	Rimoldi et al (2019)^42^
In vitro and in vivo	Evaluation of chitin–glucan complex in rats	Fungi (*Aspergillus niger*)	Prebiotic activity	Chitin–glucan promotes Bifidobacteria in rats	Alessandri et al (2019)^43^
Chitin and chitosan					
In vitro	Study of chitin and chitosan oligosaccharide (COS) impact on *Escherichia coli* TG growth	NR	Prebiotic activity	Chitin (0.5% wt/vol and 0.1% wt/vol) inhibited *Escherichia coli* growth; COS (0.05% wt/vol, 0.1% wt/vol, 0.5% wt/vol)	Selenius et al (2018)^41^

Abbreviations: NR, not reported; SCFA, short-chain fatty acid.

### Chitin Enhances Short-Chain Fatty Acid Production

The maintenance of the microbiota is a virtuous cycle of anabolism and catabolism of short-chain fatty acids (SCFAs). SCFAs are bacterial fermentation products that create an ideal growth environment for the organism (ie, Bifidobacteria and Lactobacilli).^37^ SCFAs promote the integrity and proliferation of intestinal epithelial cells, repair damaged epithelial tissue, and facilitate differentiation, exerting antitumoral effects.^38^ Chitin enhances the production of SCFAs that benefit the microbiome and seems to be linked to intestinal immunity. In fact, several immunocompetent cells express receptors for SCFAs; thus, immune homeostasis of the digestive tract may also depend on SCFA production.^39^ However, the modulatory role of chitin on the microbiota needs further investigation.

### Modulatory Effect of Chitin on the Microbiota

In vitro studies have shown a positive impact of the prebiotic activity of chitin from mealworm (*T molitor*) flour. de Carvalho et al^40^ simulated the human digestive process using a gastrointestinal simulator and performed anaerobic fecal fermentation. The results suggest that Bacteroidaceae and Prevotellacae, microorganisms with proteolytic and saccharolytic activities whose fermentation product is propionate, are increased in digested flour. However, digested flour does not retain important butyrate-producing species such as the *Clostridium coccoides*/*Eubacterium rectale* group, *Roseburia* subcluster, and *Faecalibacterium prausnitzii*, while undigested flour seems to retain them. However, chitin gastrointestinal activity is not strictly related to microbiota modulation; in fact, defensive strategies against pathogens are exhibited. Chitin and chito-oligosaccharide, a chain of few N-acetyl-d-glucosamine units, showed antiproliferative effects against *Escherichia coli* in vitro. In particular, chitin (0.5% wt/vol and 0.1% wt/vol) inhibited *E coli* growth, while chitosan oligosaccharide (COS) (0.05% wt/vol, 0.1% wt/vol, and 0.5% wt/vol) limited it.^41^

In order to obtain a more accurate picture, in vivo studies need to be improved. Among the most recent in vivo studies, the administration of insect meal, mainly *Hermetia illucens* and *Gryllodes sigillatus*, was tested. The intake of insects, also considering the relative percentage of chitin, had no negative impact on the richness and relative abundance of microorganisms. In a clinical trial, 25 g of 100% cricket powder was administered to healthy volunteers for 14 days. The microbiota showed no negative alterations and Bifidobacteria seemed to benefit. Furthermore, no inflammatory states occurred in the intestine, which suggests good tolerance of the food in the human gut.^35^

In a study on rainbow trout, different inclusions of defatted *H illucens* meal appeared to promote the *Mycoplasma* population, the most abundant genus in trout and strongly linked to SCFA production.^42^ In the poultry model, *H illucens* meal increased the richness and improved the SCFA profile. In general, chitin intake does not compromise the health status of participants and seems to play a role in modulating the microbiota, especially according to the SCFA profile data. However, several studies have shown that the prebiotic activity of chitin appears to be exacerbated by conjugation with beta-glucans.

The chitin–glucan conjugation is particularly interesting. An in vivo study in a rat model, with chitin–glucan associated with *Bifidobacterium breve* 2 L, largely promoted the population of *B breve* 2 L compared with the administration of *B breve* 2 L orally. In addition, the relative abundance of bifidobacterial species also increased when the chitin–glucan–*B breve* 2 L complex was administered.^43^ Investigating the effects of chitin–glucan, an in vitro study by Marzorati et al^44^ tested 2 different doses of chitin–glucan (1.5 g/d and 4.5 g/d) in a Simulator of the Human Intestinal Microbial Ecosystem (SHIME^®^, ProDigest and University of Ghent, Belgium) for 3 weeks. The results showed the higher dose had more positive effects on SCFAs by promoting the production of propionate and butyrate. Butyrate acts as a primary source of energy for the intestinal epithelium with protective effects against inflammation and colon cancer. Among microbial species, the growth of Bacteroidetes and Firmicutes was promoted. More specifically, the Firmicutes/Bacteroidetes ratio was lower, suggesting a predominance of Bacteroidetes. Alongside these species, an increase was also shown in *Roseburia*, which is a major producer of butyrate.

The same dose of chitin–glucan was used with healthy individuals in a clinical trial. Comparing the microbiota of volunteers before and after treatment with 4.5 g per day for 3 weeks revealed the maintenance of beta diversity, and an increase in *Roseburia* and *Eubacterium* was observed following administration. In addition, a large production of butyrate, isovaleric acid, caproic acid, and vaccenic acid was recorded, thus suggesting a growth promotion of positive species related to SCFA production. In conclusion, chitin has positive prebiotic activities without altering the health status of volunteers. However, the modulatory activity needs further investigation considering the ecological and systemic complexity of the gut microbiota. Considering the promising chitin–glucan outcomes, studies on the association with other dietary fibers could be improved.

## CHITIN, CHITOSAN, AND CANCER

### Inflammatory Modulation of Chitin on Cancer Cells

One of the most widespread concerns among people regarding chitin intake is the possible impact on health, and in particular, the possible correlation between chitin and cancer (Table 2). In recent years, many studies have investigated the effects of chitin and its derivatives, mainly chitosan, on cellular activity. Chitin constitutes the exoskeleton of insects and the shell of crustaceans, but it is also a component of the wall of fungi and bacteria. With regard to defensive mechanisms, chitin is recognized as having pathogen-associated molecular patterns (PAMPs) by activating macrophages and neutrophils that induce the enzymatic response of chitinases. Chitinases are enzymes conserved in vertebrates and also present in humans that bind and degrade chitin molecules but which are not considered pancreatic enzymes. In fact, chitinase expression is a consequence of the activity of immune system cells and proinflammatory stimuli. Chitinase-like proteins (CLPs) are an interesting class of chitinases for the study of carcinogenesis. These enzymes bind chitin but do not have chitinolytic activity, are upregulated during inflammation or cancer, influence the inflammatory response using pathways such as interleukin (IL)-3 (IL-3)–mediated signaling and mitogen-activated protein kinase (MAPK) signaling, and appear to be involved in cell proliferation, cell survival, and angiogenesis.^45^ An in vivo study by Libreros et al^46^ attempted to clarify the correlation between chitinase-like proteins, chitin, and cancer by administering an intraperitoneal chitin treatment to mice with mammary tumors.

**Table 2.** In Vivo and In Vitro Studies on the Correlation of Chitin and Chitosan with Cancer

Molecule and assay	Study	Source	Activity	Description	Reference
Chitin					
In vivo	Evaluation of chitin effects on mice affected by breast cancer	Crustaceans	Anticancer activity	Chitin (1 mg/mouse every 3 d for 5 wk) reduced cancer growth by modulating the immune system	Libreros et al (2012)^46^
In vitro	Impact of chitin hydrolysate on human breast cancer cells	NR	Angiogenic activity	Induction of VEGF-C expression and promotion of cell migration	Timoshenko et al (2011)^47^
Chitosan					
In vivo	Chitosan oligosaccharide administered via os in mice affected by colon rectal cancer	NR	Anticancer activity	Chitosan oligosaccharide 100 mg/kg and 500 mg/kg reduces cancer by about 60%. Chitosan oligosaccharide activates AMPK signaling and inhibits the early stages of carcinogenesis.	Mattaveewong et al (2016)^53^
In vitro	Cell culture–based assessment of antitumor activity of chitosan	Shrimp	Anticancer activity	Chitosan reduced cell adhesion of A375 capacity. In the SK-MEL-28 cell line, chitosan increased the levels of apoptosis, which in the RPMI 7951 line is exacerbated in a concentration-dependent and time-dependent manner. Increased levels of CD95/Fas receptors were detected on the cell surface of the RPMI-7951 line when exposed to chitosan, suggesting an increased sensitivity of the cells to the induction of apoptosis via Fas receptor.	Gibot et al (2015)^49^
	Study on the cytotoxicity and antiproliferative activity of different molecular-weight chitosan on different cancer cell lines	Insects (mayfly)	Anticancer activity	Concentrations of 250 μg/mL showed inhibitory activity on cancer cells (ie, A549 and WiDr)	Tan et al (2018)^52^
	Study on cancerous cell lines in order to evaluate cytotoxic activity of chitosan	NR	Anticancer activity	Low- and high-molecular-weight chitosan at a concentration of 1 mg/mL exhibited cellular apoptosis of cancer cell lines represented by MCF7, Saos-2, HeLa cells	Abedian et al (2019)^51^
	Evaluation of cytotoxic properties of chitosan obtained from mushroom species	Mushroom species (*Boletus bovinus*, *Laccaria laccata*)	Cytotoxic effect	Chitosan films and solutions, characterized by low molecular weight and a high degree of deacetylation, showed cytotoxic effects on cancerous hepatoma and normal ovary cells (MH-22A and CHO respectively).	Oberemko et al (2019)^48^
	Effects of complex chitosan selenate on lung cancer cells	NR	Anticancer activity	Chitosan selenate exhibited little cytotoxicity to normal lung MRC-5 cells in vitro, indicating that it may be a useful anti-cancer agent for the treatment of lung cancer.	Gao et al (2020)^50^
Chitin and chitosan					
In vitro	Evaluation of cytotoxic effect of chitin and chitosan on Hep2 and RD cell lines	Shrimp	Anticancer activity	Chitin (60% DD-350 kDa) was cytotoxic at IC_50_ = 400 μg/mL and 200 μg/mL against Hep2 cells and RD cells lines, respectively. The lowest IC_50_ was attributed to chitosan (39% DD-20 kDa), 300 μg/mL in Hep2 and 190 μg/mL in RD	Bouhenna et al (2015)^56^
In vitro	Evaluation of anticancer activity of chitin and chitosan against human ovarian cancer cell line	Shrimp shells of *Penaeus monodon*	Anticancer activity	Chitin concentration of 50 μg/mL showed the ability to suppress the growth of PA-1 tumor cells. Chitosan (10 μg/mL) showed the potential to suppress 100% of the growth of PA-1 tumor cells.	Srinivasan et al (2018)^57^

Abbreviations: AMPK, AMP-activated protein kinase; IC_50_, half-maximal inhibitory concentration; NR, not reported; RPMI, Roswell Park Memorial Institute; VEGF-C, vascular endothelial growth factor C.

From the literature, the CLP Chi3L-1 seems to be particularly expressed in human cancer. In a mouse model, the enzyme was detected in the plasma of affected mice with the induction of macrophages to release cytokines as chemokine (C-C motif) ligand 2 (CCL2), chemokine (C-X-C motif) ligand 2 (CXCL2) and matrix-degrading enzymes (MMP-9), and in addition, interferon-gamma (IFN-γ) levels were lower. Mice treated with chitin doses of 1 mg/mouse showed a decrease in proinflammatory cytokines and an increase in IFN-γ. Chitin was shown to indirectly modulate the inflammatory state by acting on the immune system with anti-tumorigenic effects. The response induced by chitin is T-helper 1 cells (TH1): chitin mediated macrophages through a mechanism involving IL-12. The other hypothesis is related to chitin size. The chitin used in the study was small (1–10 µm), and it could be possible that chitin was bound and degraded by chitinases and initiated a TH1-type immune response. However, the correlation between chitin size and biological effects still needs to be investigated. In a study by Timoshenko,^47^ chitin hydrolysate, a highly concentrated form of chitin, appeared to promote the expression of the vascularization factor vascular endothelial growth factor C (VEGF-C) on the MDA-MB-231 (human breast cancer) cell line. Angiogenesis is a very complex process that supports cell and tissue proliferation; however, high values of vascularization factors are indirect indicators of carcinogenesis. Thus, Timoshenko^47^ reported that chitin was not directly related to tumor occurrence, and the author provided no information regarding the composition and size of the chitin hydrolysate used that could influence the outcome. However, among the interesting results reported in the study, chitin hydrolysate did not alter the morphology of cells expressing high levels of VEGF-C, suggesting that it has no cytotoxic effect.

### Chitosan Involvement in Apoptosis and Cytotoxicity in Cancer Cells

In the same way that the structure, composition, and size of chitin may have different biological effects, a similar argument applies to its derivatives. In particular, chitosan, the deacetylated form of chitin, has been widely studied for its antitumor effects. However, Oberemko et al^48^ demonstrated that chitosan from fungi *Boletus bovinus* and *Laccaria laccata* could induce apoptosis and necrosis-like effects both for MH-22A (cancerous mouse hepatoma) and CHO (non-cancerous Chinese hamster ovary) cells. In this latter study, low-molecular-weight chitosan at a high grade of deacetylation was used at high concentrations. The chitosan solution may have affected the outcome on cell lines, suggesting a possible low cellular tolerance to high concentrations. In fact, other studies have shown different results. Gibot et al^49^ demonstrated that the incubation of a 2-mg/mL solution of chitosan deacetylated in 0.1% acetic acid with A375, SK-MEL-28, and Roswell Park Memorial Institute (RPMI) 7951 cell lines (melanoma cancer cells) induced a decrease in viability, growth, and proliferation rate. In addition, chitosan solution led to apoptosis in the SK-MEL-28 and RPMI-7951 lines but not in the A375 line. In contrast, it did not induce apoptosis in healthy human cells taken as a control (primary dermal fibroblasts).

Exactly how chitosan induces apoptosis in melanoma cells is still unclear, and it is assumed that it could be inducted through mitochondrial pathways. The balance between Bcl-2 and Bax determines a pro- or anti-apoptotic process. In the case of the RPMI-7951 lineage, chitosan was shown to upregulate the pro-apoptotic protein Bax and downregulate the anti-apoptotic Bcl-2. In addition, high levels of CD95/Fas receptors were detected on the cell surface of the RPMI-7951 line after exposure to chitosan, which suggests that the cells were more sensitive to the induction of apoptosis via the Fas receptor. In contrast, the A375 line maintained its anti-apoptotic state despite incubation with chitosan but decreased its adhesive capacity. For the SK-MEL-28 line, apoptosis levels remained low, probably due to the slowing of the proliferation rate and, consequently, of tumor growth. The antitumor potential of chitosan has also been studied in combination with other components able to counteract cancer.

Gao et al^50^ investigated the apoptosis processes induced by a chitosan-selenium compound on A549 lung cancer cells. The expression of the Fas/FasL apoptotic pathway in A549 cells was shown to increase after the treatment. The survival rate of A549 cells was significantly lower than human normal lung MRC-5 cell lines used as the control, and the growth inhibition occurred in a dose-dependent manner. Also in human breast cancer (MCF-7), human cervical epithelial cancer (HeLa) and osteosarcoma (Saos-2) cell chitosan exhibited antitumoral effects.^51^ The authors tested 2 molecular-weight variants of chitosan: low molecular weight (100–300 kDa) and high molecular weight (600–800 kDa). In line with other studies, after treatment with chitosan, cytotoxic effects decreased the viability of the cancerous cell lines in a dose-dependent manner. In fact, increasing levels of chitosan (2 mg/mL) reduced cell viability by up to 70%–90% in cancerous cells. In contrast, dermal fibroblasts were not heavily affected.

The specificity of the chitosan effect seems to be dependent on the cell charge and interaction mechanisms at the cellular level. This could be a result of its cationic polyelectrolyte character due to the positive charges of the amine groups in the d-glucosamine units leading to electrostatic interaction with negatively charged molecules. These interactions between negatively charged groups of cancer cells and the positively charged groups of chitosan may result in a cytotoxic effect. In contrast to other studies that report a size-dependent effect of chitosan, in the Abedian et al^51^ experiment, low and high molecular weights of the molecule resulted in no significant difference in antiproliferative activity, except for the concentration of 2 mg/mL on MCF-7 and 4 mg/mL on fibroblasts.

Chitosan apoptotic activity was also demonstrated in an in vitro study on HeLa (human cervical cancer), A549 (human lung adenocarcinoma) and WiDr (colon adenocarcinoma) lines with commercial chitosan and chitosan recovered from mayfly, an aquatic insect of the Ephemeroptera order.^52^ Commercial chitosan was treated to obtain different molecular weights in order to distinguish between low molecular weight, medium molecular weight, and high molecular weight. The effects of chitosan extracted from insects were compared with commercial chitosan. The outcomes showed that, at increasing concentrations of chitosan (from 25 up to 500 µg/mL), chitosan had cytotoxic effects on the non–tumor cell line of L929 only at the high concentration (500 µg/mL) in cell culture.

Results on the evaluation of the apoptotic effects of chitosan polymers are particularly interesting. Surprisingly, the effects seemed to depend on 2 main factors, the molecular weight and the cell line itself. The cell viability of the HeLa line did not respond to 250 µg/mL, which had been previously assessed as a nontoxic chitosan concentration for the non-tumor lines. In contrast, the A549 and WiDr lines appeared to be influenced by chitosan from mayfly and by commercial chitosan at low molecular weights. Chitosan molecules can exhibit different effects depending on their chemical-physical characteristics, such as their molecular weight. In an in vivo study, COS, a molecule obtained by the hydrolysis and deacetylation of chitin, was evaluated in colorectal cancer–induced mice.^53^

According to the literature, COS with a molecular weight close to 5000 Da inhibited the intestinal inflammatory state associated with mucosal damage.^54^ For this reason, COS was used in vivo to evaluate its effects on colorectal cancer. Three doses of COS (20 mg/kg, 100 mg/kg, and 500 mg/kg) were administered by gavage to mice with colorectal cancer, and a dose-dependent reduction in the tumor size was noted, especially following the 100-mg/kg and 500-mg/kg administrations. Chitosan oligosaccharide appeared to inhibit the nuclear factor–κB (NF-κB) signaling and mammalian target of rapamycin (mTOR) signaling typically involved in the early stages of tumor development. Chitosan oligosaccharide also inhibited the expression of MMP-9 implicated in advanced tumor status, thus suggesting a delay in disease progression. However, 500 mg/kg is the dose that has shown chemoprotective properties, compared with a human dose of 2–3 g per day taken for 24 weeks without adverse effects.^55^

Several studies have compared the cytotoxic effects of chitin and chitosan on tumor cell lines. In contrast to chitin, chitosan contains positively charged groups that may interact with negatively charged groups on the cell membrane, resulting in damage to cancerous cells. In an in vitro study involving 2 human cancer cell lines (ie, a human embryo rhabdomyosarcoma cell line [RD] and human larynx carcinoma cell line [Hep2]), chitosan showed cytotoxic activity of tumor cell lines at lower half-maximal inhibitory concentration (IC_50_) values compared with chitin. Specifically, the growth of Hep2 cells was inhibited with IC_50_ values of 400 μg/mL and 300 μg/mL for chitin and chitosan, respectively.^56^ Lower IC_50_ values were obtained for RD cell lines (200 μg/mL of chitin and 190 μg/mL of chitosan). These data suggest that chitosan has greater cytotoxic efficacy than chitin in a PA-1 human ovarian cancer cell line. In this latter case, chitin concentrations of 50 μg/mL suppressed the growth of PA-1 tumor cells and 10 μg/mL of chitosan was sufficient for the same potential of cell suppression.^57^

In conclusion, studies that have reported the antitumor effects of chitin are mainly in vitro. There have been few in vivo studies and more are needed. In contrast, chitosan has been reported in in vitro and in vivo studies to consistently demonstrate anti-inflammatory and antitumor activities, including when recovered from other sources such as insects. However, chitosan has a wide variety of chemical-physical characteristics that should be analyzed in detail.

## CONCLUSION

In vitro and in vivo studies on prebiotic activities have shown the possible microbiota modulation of chitin and its important communication with the immune system. With regard to a possible link between chitin and cancer, no in vitro studies have confirmed its cancerogenic activity. In contrast, many recent in vitro studies suggest a cytotoxic effect of chitin on cancer cell lines. Chitosan, the deacetylated form of chitin, has also been studied for the same antitumor properties. It exhibits important apoptotic and cytotoxic activities against several cancer cell lines without affecting the noncancerous cells.

In conclusion, the literature has so far not shown a direct connection of chitin and chitosan with the onset of cancer, whereas cytotoxic and apoptotic activities against cancerous lines have been demonstrated.

## Data Availability

This review is based on the reanalysis of data available in published articles and public datasets, as referenced throughout the manuscript. No new data were generated.
